# Chloroform and Ether

**Published:** 1873-12

**Authors:** 


### Chloroform and Ether.

Our Boston friends seem determined to prove to the world that Ether is the only safe and reliable anæsthetic, and occasionally go to extremes, which to us poor mortals who live at the circumference of the wheel seems a little strange. We copy the following verdict of a Boston Jury upon a case of death from a mixture of Chloroform and Ether. The jury found:

That Mary F. Crie came to her death on Monday, the 10th day of November, 1873, between eleven A. M. and one P. M., in the office of Dr. Charles Eastham, a dentist, No. 25 Tremont street, Boston, and that her death was caused by the inhalation of chloroform administered in a mixture of chloroform and ether by the said Dr. Eastham. The jury use this opportunity tc caution the public against the inhalation of so dangerous an agent as chloroform for the production of insensibility to pain. In the opinion of the jury the inhalation of sulphuric ether is safe, while the inhalation of chloroform either alone or miqed, is always attended with danger.

It was signed by Ezra Palmer, M. D., John A. Lanson, M. D., George Fabyan, M. D.. George Lotz, M. D., Thomas Restieaux and Thomas Doliver.

The Boston jury here seem to ignore the fact that ether is capable of producing death, but wisely announce that chloroform alone was its cause. While we heartily agree with the gentlemen that ether is by far the safest anæsthetic, we think that occasions are not unheard of in which it was apparently the cause of death, and are at a loss to ascertain the course of reasoning pursued by the jury which enabled them to arrive at the conclusion that the chloroform contained in the mixture, and not the ether or both combined produced the fatal result.

—We learn from the *Philadelphia Medical Times* that the novel operation for Aneurism by Dr. Lewis, to which we alluded some time since, resulted fatally.

—The regular meeting of the Erie County Medical Society will be held in this city on the first Tuesday in January, 1874.

